# Endoscopic ultrasound with double-balloon endoscopy for the diagnosis of inverted Meckel’s diverticulum: a case report

**DOI:** 10.1186/1752-1947-6-328

**Published:** 2012-09-28

**Authors:** Akihiro Araki, Kiichiro Tsuchiya, Shigeru Oshima, Eriko Okada, Shinji Suzuki, Junko Morio Akiyama, Toshimitsu Fujii, Ryuichi Okamoto, Mamoru Watanabe

**Affiliations:** 1 Department of Gastroenterology & Hepatology, Graduate School of Medicine, Tokyo Medical and Dental University, 1-5-45 Yushima, Bunkyo-ku, Tokyo 113-8510, Japan

**Keywords:** Double-balloon endoscopy, Endoscopic ultrasound, Inverted Meckel’s diverticulum

## Abstract

**Introduction:**

Inverted Meckel’s diverticulum has usually been misdiagnosed in the cases based on computed tomography images presented in the literature. The final diagnosis was made intra-operatively or by pathology reports after surgery. Despite this, preoperative diagnosis could be made successfully by using endoscopic ultrasound with double-balloon endoscopy prior to surgery.

**Case presentation:**

A 60-year-old Japanese woman with severe anemia complained of several episodes of black stool over the preceding 2 years. Abdominal computed tomography showed a 3.0-cm low-density tumor in the ileum, suggesting a diagnosis of intestinal lipoma. Examination of the tumor by endoscopic ultrasound with double-balloon endoscopy revealed a hypo-echoic layer corresponding to the muscularis propria, and a hyper-echoic layer corresponding to the fat tissue. These findings, which suggested that the tumor included areas outside the intestinal serosa, are not typical for a lipoma, despite the existence of a hyper-echoic layer corresponding to fatty tissue. We then considered a diagnosis of inverted Meckel’s diverticulum.

**Conclusion:**

Lipoma and inverted Meckel’s diverticulum are difficult to differentially diagnose by computed tomography. Polypectomy is the preferred therapeutic approach when a lipoma is present; however, polypectomy in a patient with Meckel’s diverticulum requires full-thickness resection. Situations where polypectomy is performed without preparing for full-thickness resection can be avoided by first making a precise diagnosis using double-balloon endoscopy and endoscopic ultrasound.

## Introduction

Inverted Meckel’s diverticulum has usually been misdiagnosed in the cases based on computed tomography (CT) images presented in the literature [1]. The final diagnosis was made intra-operatively or by pathology reports after surgery. Despite this, preoperative diagnosis could be made successfully by using endoscopic ultrasound (EUS) with double-balloon endoscopy (DBE) prior to surgery.

## Case presentation

A 60-year-old Japanese woman with severe anemia complained of several episodes of black stool over the preceding 2 years. Upper and lower gastrointestinal tract endoscopies were performed at another hospital and the findings were unremarkable. She was referred to our hospital for further evaluation. Blood tests confirmed the known anemia (hemoglobin 7.2g/dL; normal range 13.0–15.5g/dL) with iron deficiency (iron 12μg/dL; normal range 50–140μg/dL).

**Figure 1:**
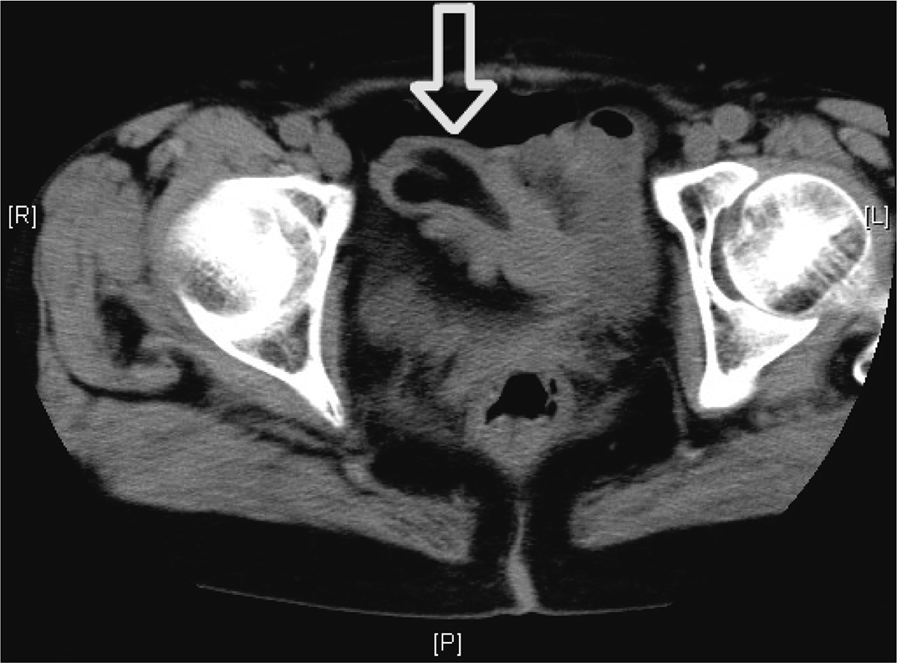
**Abdominal computed tomography. **Abdominal computed tomography showed a 3.0-cm low-density tumor in the ileum, suggesting a diagnosis of intestinal lipoma.

An abdominal CT showed a 3.0-cm low-density tumor in the ileum, indicative of an intestinal lipoma (Figure 1). Intermittent episodes of melena continued after admission, leading us to perform retrograde DBE to enable diagnosis and treatment.

**Figure 2:**
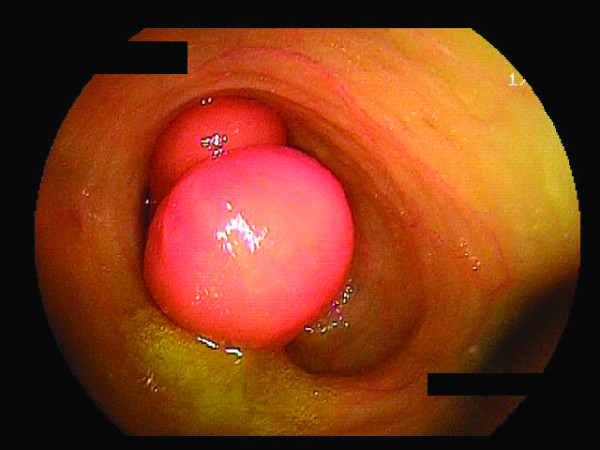
**Double-balloon endoscopy. **Double-balloon endoscopy images displayed a dumbbell-shaped tumor (30 × 35mm) on the anti-mesenteric side of the ileum, approximately 85cm from the ileocecal valve.

**Figure 3:**
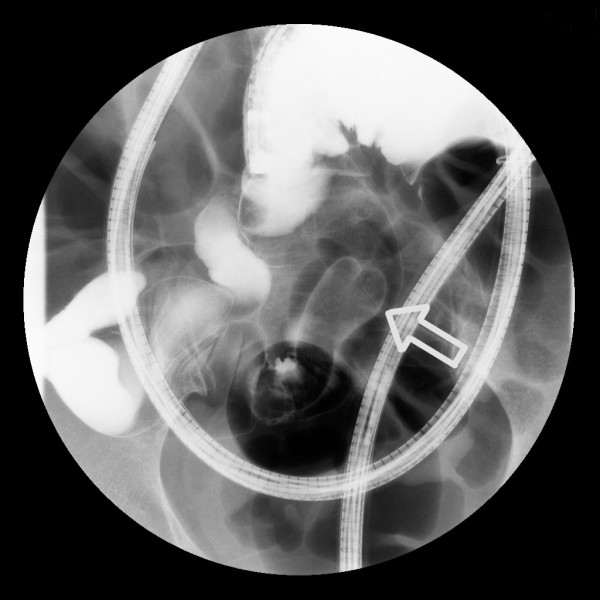
**Selective contrast-enhanced radiography. **A dumbbell-shaped tumor was shown by selective contrast-enhanced radiography.

**Figure 4:**
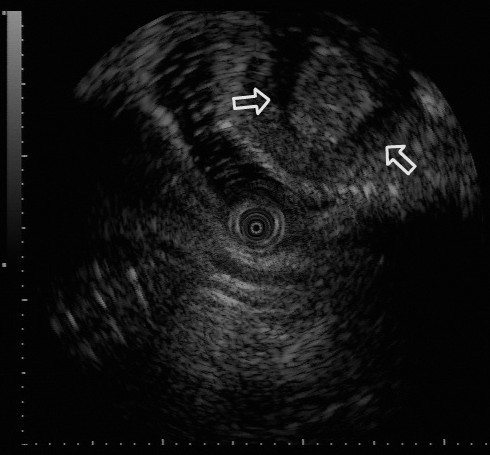
**Endoscopic ultrasound examination. **Endoscopic ultrasound examination of the tumor revealed a hypo-echoic layer corresponding to the muscularis propria and a hyper-echoic layer corresponding to fat tissue.

**Figure 5:**
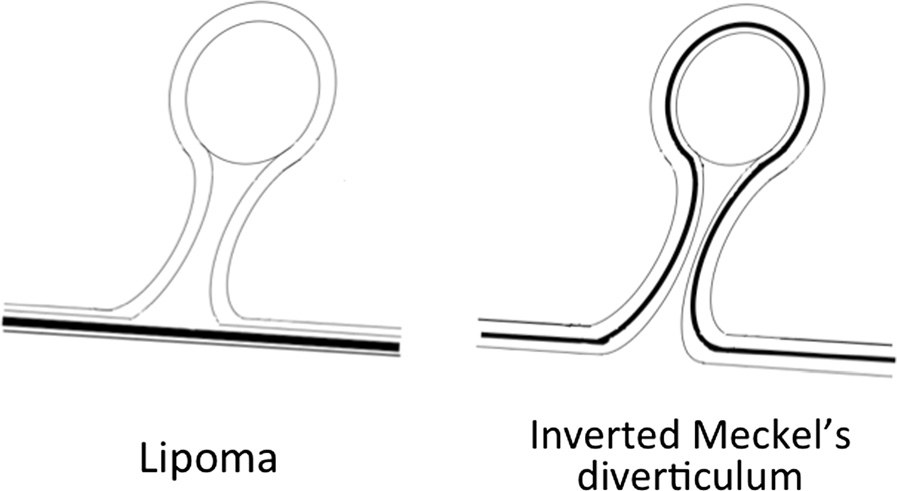
**Illustration of the relationship between the muscularis propria and serosa. **In the inverted Meckel’s diverticulum, muscularis propria and serosa exists in the inside of the protruded lesion

DBE images displayed a dumbbell-shaped tumor (30 × 35mm) on the anti-mesenteric side of the ileum, approximately 85cm from the ileocecal valve (Figure 2). A similar finding was shown by selective contrast-enhanced radiography (Figure 3). The cushion sign was detected when the biopsy forceps were pushed into the tumor. This sign usually confirms the diagnosis of intestinal lipomas. EUS revealed a hypo-echoic layer corresponding to the muscularis propria and a hyper-echoic layer corresponding to the fat tissue (Figures 4 and 5). These findings, which suggested that the tumor included areas outside the intestinal serosa, are not typical for a lipoma**,** despite the existence of a hyper-echoic layer corresponding to fatty tissue. Based on these findings inverted Meckel’s diverticulum was suspected as possible diagnosis and laparoscopic surgery was considered to be the appropriate treatment. A surgical specimen showed ectopic pancreatic tissue around the top of a tumor-like elevation, thus confirming our diagnosis.

The postoperative course was uneventful and the patient was discharged with an improvement in anemia.

## Discussion

Lipoma and inverted Meckel’s diverticulum are difficult to differentially diagnose by CT. Their main difference is the existence of serosa inside of the polyp. However, if a polypectomy were to be performed in a patient with inverted Meckel’s diverticulum, a full-thickness resection should be necessary. The accurate preoperative diagnosis of inverted Meckel’s diverticulum by DBE and EUS could avoid unnecessary polypectomies without preparation for full-thickness resection and the subsequent complications. The diagnostic characteristics of Meckel’s diverticulum in this case included a tumor located between 70 and 100cm from the ileocecal valve on the anti-mesenteric side, and EUS findings of a hyper-echoic layer encompassing areas outside the intestinal serosa. This case is a typical example of inverted Meckel’s diverticulum diagnosed by DBE and EUS, and so an unforced polypectomy was avoided. The limitation of DBE is that it cannot be used without the agreement of the patient [2,3]. We specify the indications of EUS to evaluate the depth of region in the intestinal wall and to prove the existence of the serosa in the polyp.

## Conclusion

DBE is an innovative method that facilitates disease treatment and enables pathological assessment by biopsy throughout the entire small intestine [4]. DBE can be used as an alternative diagnostic tool in cases where video capsule endoscopy is contraindicated due to the characteristics of the ileus or diverticulum. DBE is a powerful tool for intestinal treatment and an alternative to laparotomy. In this case, DBE and EUS revealed the existence of serosa in the polyp, which means that the polyp includes the extraluminal adipose tissue. Meckel’s diverticulum can be easily diagnosed by recognizing the double-lumen sign (composed of true lumen and an anti-mesenteric-sided Meckel’s diverticulum) during DBE.

The combination of DBE and EUS can diagnose an inverted diverticulum that appears to be a polyp.

## Consent

Written informed consent was obtained from the patient prior to publication of this case report and accompanying images. A copy of the written consent is available for review by the Editor-in-Chief of this journal.

## Competing interests

The authors have no financial or other interests in the manufacture or distribution of any equipment, device**,** or drug mentioned in this article. The authors declare that they have no competing interests.

## Authors’ contributions

AA, KT, SO, EO, SS, JMA and TF performed the DBE**;** AA was a major contributor to the writing, analysis, and interpretation of patient data for gastrointestinal bleeding. RO and MW were major contributors to the writing and proofreading of the manuscript. All authors read and approved the final manuscript.
